# Evaluating the Role of Artificial Intelligence in Making Clinical Decisions for Treating Acute Pancreatitis

**DOI:** 10.3390/jcm14124347

**Published:** 2025-06-18

**Authors:** Mete Ucdal, Amir Bakhshandehpour, Muhammed Bahaddin Durak, Yasemin Balaban, Murat Kekilli, Cem Simsek

**Affiliations:** 1 Division of Internal Medicine, Faculty of Medicine, Hacettepe University, Ankara 06800, Türkiye; meteucdal@hacettepe.edu.tr (M.U.); a.bkhshandhp@gmail.com (A.B.); bdurak@hacettepe.edu.tr (M.B.D.); ybalaban@hacettepe.edu.tr (Y.B.); drkekilli@gmail.com (M.K.); 2 Faculty of Medicine, Hacettepe University, Ankara 06800, Türkiye; 3 Division of Gastroenterology, Faculty of Medicine, Hacettepe University, Ankara 06800, Türkiye; 4 Division of Gastroenterology, Faculty of Medicine, Gazi University, Ankara 06800, Türkiye

**Keywords:** clinical decision support, acute pancreatitis, artificial intelligence, natural language processing, pancreatic necrosis, pancreatic pseudocyst, enteral nutrition, assessment of pancreatitis severity

## Abstract

**Background/Objectives:** Acute pancreatitis (AP) is an illness that requires prompt diagnosis and treatment since it has the potential to become life-threatening. The American College of Gastroenterology 2024 (ACG24) guidelines offer a framework for diagnosis, severity, and treatment criteria. To assess Google Gemini application of ACG24 guidelines to Medical Information Mart for Intensive Care-III AP cases for risk, nutrition, and complication management. **Methods:** This observational cross-sectional study was based on 512 patients with AP who were treated in the Medical Information Mart for Intensive Care-III database from 2001 to 2012. The study compared the efficiency of Gemini in relation to the ACG24 guidelines in the three main areas of risk stratification, enteral nutrition timing, and necrotizing pancreatitis management. Enteral nutrition, according to the ACG24 guidelines, should be started within 48 h for patients who are capable, and antibiotics should only be used for confirmed infected necrosis. **Results:** The study included 512 patients who were divided into two groups: 213 patients with mild pancreatitis (41.6%) and 299 patients with severe pancreatitis (58.4%). The model achieved 85% accuracy for mild cases and 82% accuracy for severe cases of pancreatitis. The Acute Physiology and Chronic Health Evaluation II and Ranson scores matched the predictions of Gemini for both mild cases (*p* = 0.28 and *p* = 0.33, respectively) and severe cases (*p* = 0.31 and *p* = 0.27, respectively). The recommendations for early enteral nutrition and delayed feeding in mild cases were correct for 78% of patients, but the system suggested oral intake prematurely in 8% of severe cases. The antibiotic guideline compliance reached 82% among 156 patients with necrotizing pancreatitis, and the procedure for draining infected necrosis was correct 85% of the time. **Conclusions:** The Gemini model achieved 78–85% accuracy in determining pancreatitis severity and adherence to treatment guidelines but showed lower accuracy in nutrition timing compared to other parameters. Core Tip: This study evaluated the Google Gemini model in applying the American College of Gastroenterology 2024 guidelines for acute pancreatitis across 512 Medical Information Mart for Intensive Care-III cases. Results demonstrated 85% accuracy in severity classification, precise prediction of Acute Physiology and Chronic Health Evaluation II and Ranson scores, and 78–85% compliance with nutritional and necrotizing pancreatitis management guidelines. These findings suggest that artificial intelligence-based clinical decision support systems can provide rapid, consistent, and guideline-concordant recommendations, which are particularly valuable in settings with limited specialist expertise.

## 1. Introduction

Acute pancreatitis (AP) is one of the leading gastrointestinal conditions requiring hospital admission. It affects 300,000 patients yearly in the United States while generating healthcare expenses above USD 2.5 billion [1]. In 2024, the American College of Gastroenterology (ACG) published updated evidence-based guidelines for managing AP (ACG24). The guidelines provide comprehensive recommendations for diagnosis, risk stratification, nutritional support, and management of complications [2].

The development of artificial intelligence in healthcare has brought large language models (LMMs) into consideration as promising tools for supporting clinical decisions. The text-based nature of medical information and communication makes these models suitable for assisting diagnosis and treatment planning. The deployment of these models in complex medical conditions requires a thorough evaluation of their reliability and guideline adherence before implementation. AP is an optimal testing condition for the evaluation of artificial intelligence (AI) in clinical decisions because it demands a precise risk evaluation, immediate clinical responses, and well-defined treatment protocols [3].

This study evaluates three critical management domains from the American College of Gastroenterology 2024 guidelines: severity risk stratification, timing of enteral nutrition, and management of necrotizing pancreatitis. These parameters were selected because they represent the guideline’s Grade A recommendations—interventions with the strongest evidence base demonstrating a significant impact on patient mortality, length of hospital stay, and complication rates. Severity stratification guides triage decisions and monitoring intensity; early enteral nutrition has been proven to reduce infectious complications by 50% and mortality by 30%; and appropriate management of necrotizing pancreatitis, particularly when infected, directly determines survival outcomes. We employed Google’s Gemini 1.5 Pro model through Vertex AI for two critical reasons. First, this model demonstrates advanced clinical reasoning capabilities necessary for interpreting complex medical data. Second, deployment via Vertex AI ensures compliance with the PhysioNet Credentialed Data Use Agreement, as this infrastructure processes data without retention for model training—a requirement that excludes most consumer-facing language model services from consideration when handling protected health information. Prior AI research in acute pancreatitis has largely taken two limited approaches. First, standalone machine-learning models—such as gradient-boosted decision trees (e.g., XGBoost) and random forests—have been trained to predict disease severity or specific outcomes (e.g., ICU admission) with high discriminative performance (AUCs ≥ 0.90) using admission variables and classical scores like APACHE II or C-reactive protein [4]. Radiomics and convolutional neural network approaches applied to CT imaging have achieved AUCs of ~0.75–0.78 for classifying mild versus severe cases, outperforming Glasgow and BISAP criteria in single-center cohorts [5]. However, these models remain narrow in scope, providing predictions without integrating the sequential, guideline-driven actions (e.g., the timing of enteral feeding or drainage procedures) required in real-world clinical workflows. Second, large language models (LLMs) such as ChatGPT-3.5 and GPT-4 have been evaluated on theoretical Q&A tasks derived from AP guidelines, achieving accuracies from ~59% to 94% on multiple-choice and short-answer queries [6]. Despite correctly recalling broad recommendations, these studies did not test LLMs on actual patient records or measure real-case guideline adherence; they also noted issues with ‘hallucinated’ references and generalized responses lacking clinical nuance. This fundamental gap—between isolated predictive models and theoretical knowledge testing—highlights the absence of comprehensive, guideline-based decision support systems capable of processing real patient data. Our study directly addresses this limitation by evaluating LLM performance on actual clinical cases requiring sequential, evidence-based management decisions.

## 2. Materials and Methods

### 2.1. Patient Selection and Study Design

This observational cross-sectional retrospective study evaluated state-of-the-art LLMs for AP severity classification and management recommendations in alignment with clinical guidelines. This study used the MIMIC-III database (PhysioNet, Massachusetts Institute of Technology, Cambridge, MA, USA), which contains de-identified health information from more than 40,000 patients who received critical care at Beth Israel Deaconess Medical Center Beth Israel Deaconess Medical Center, (330 Brookline Avenue, Boston, MA, USA) from 2001 to 2012 [7]. Patient selection was conducted by querying the MIMIC-III database for cases of acute pancreatitis (AP) using a combination of ICD-9 codes (577.0, K85.1), laboratory values (lipase/amylase elevation ≥3× upper limit of normal), and clinical documentation of cardinal symptoms (epigastric pain, nausea/vomiting).

The evaluation of the LLM was conducted using Google Gemini (Google LLC, 1600 Amphitheatre Parkway, Mountain View, CA, USA), accessed via Vertex AI (Google Cloud Platform, Google LLC, 1600 Amphitheatre Parkway, Mountain View, CA 94043, USA) on the Google Cloud Platform, following responsible use guidelines for MIMIC-III data in online services. In accordance with PhysioNet Credentialed Data Use Agreement requirements, which explicitly prohibit sharing data with third parties, we selected Google’s Gemini model via Vertex AI on the Google Cloud Platform. This choice was made because Gemini on Vertex AI does not use prompts or responses as data to train its models and fully complies with PhysioNet’s Credentialed Data Use Agreement requirements. Our research design aimed to fully assess how the Gemini 1.5 Pro model processed clinical data while determining disease severity and generating management suggestions that align with the ACG24 guidelines for AP.

### 2.2. Time Consideration Between Data and Guidelines

While the MIMIC-III data (2001–2012) predates the ACG24 guidelines, the fundamental pathophysiology and treatment principles for AP have remained consistent over time. The core concepts in the 2024 guidelines—severity assessment using validated scoring systems, early enteral nutrition, and antibiotic stewardship for confirmed infections only—were also present in earlier guidelines, although with some refinements. To address this temporal gap, we performed a supplementary analysis comparing Gemini’s recommendations against the 2013 AP guidelines that were contemporary with the data.

### 2.3. Model Evaluation

For each case, a clinical summary containing the relevant patient information was presented to the Gemini 1.5 Pro model. The model was then asked about the severity assessment of AP and the appropriate management strategy for the patient. The responses were recorded verbatim for subsequent analysis. For each case, a comprehensive clinical summary was presented to the Gemini model in a standardized format containing the following: patient history (20%), physical examination findings (15%), laboratory data (30%), imaging results (20%), and treatment/intervention details (15%). This format reflected the actual clinical data available to practitioners and maintained consistency across all evaluated cases. The evaluation of Gemini’s recommendations was conducted through a rigorous, structured assessment protocol by two board-certified gastroenterologists with subspecialty training in pancreatology (CS with 8 years of experience and MK with 7 years of experience). The evaluation process followed a standardized methodology to ensure consistency and reliability of assessments.

For each of the 512 cases, the evaluators independently reviewed Gemini’s recommendations across four primary domains: (1) severity classification accuracy based on ACG24 criteria, (2) appropriateness of nutritional management timing, (3) antibiotic therapy decisions for necrotizing pancreatitis, and (4) timing of interventional procedures. Each evaluator utilized a structured assessment form with predefined criteria derived directly from the ACG24 guidelines. The form employed a binary scoring system where recommendations were classified as either “guideline-concordant” (score = 1) or “guideline-discordant” (score = 0), with mandatory documentation of the specific guideline section supporting each assessment.

The evaluation process proceeded in three phases. During Phase 1 (Calibration), both evaluators jointly reviewed 25 randomly selected cases to establish consensus on the interpretation of guideline criteria and ensure uniformity in the assessment methodology. Phase 2 (Independent Evaluation) involved each evaluator independently assessing all 512 cases without consultation, with evaluations completed over a four-week period to minimize fatigue-related errors. In Phase 3 (Reconciliation), all cases with discordant ratings were identified and subjected to consensus review.

Inter-rater reliability was assessed using Cohen’s kappa coefficient, which demonstrated substantial agreement (κ = 0.84, 95% CI: 0.81–0.87) across all evaluation domains. The domain-specific kappa values were the following: severity classification (κ = 0.88), nutritional management (κ = 0.82), antibiotic therapy (κ = 0.83), and intervention timing (κ = 0.85).

For the 67 cases (13.1%) where initial disagreement occurred, the two evaluators conducted joint review sessions. During these sessions, each evaluator presented their rationale with specific reference to guideline text and patient data. Consensus was achieved through collaborative discussion in 65 of 67 cases (97.0%). For the remaining 2 cases, a third senior gastroenterologist (YB with 20 years of experience and expertise in pancreatic diseases) was available to provide binding arbitration, though ultimately, this was not required, as consensus was reached through extended discussion and careful re-examination of the clinical data and guideline specifications.

All evaluation data were recorded in a secure REDCap database with built-in validation rules to ensure data integrity. The evaluation process included random quality checks, where 10% of cases were re-evaluated by both reviewers three months after initial assessment, demonstrating high intra-rater reliability (evaluator 1: κ = 0.91; evaluator 2: κ = 0.89). This comprehensive evaluation methodology ensures that our assessment of Gemini’s performance represents a robust and clinically meaningful analysis of its guideline adherence capabilities.

### 2.4. Dynamic Assessment of Model Performance

To address concerns regarding real-time decision-making capabilities, we analyzed a subset of 75 cases with data available at multiple time points during hospitalization. This enabled assessment of the model’s ability to adapt recommendations as the clinical situation evolved, simulating the dynamic nature of AP management. For these cases, we extracted clinical data at admission, 48 h, 72 h, and at 7 days (when available) to evaluate how Gemini modified its recommendations based on changing clinical parameters, laboratory values, and imaging findings throughout the disease course. This approach allowed us to better approximate real-world clinical decision-making where management strategies evolve with the patient’s condition. In addition to ACG24 guidelines, we also evaluated Gemini’s performance against the revised Atlanta Classification (2012) and UK Working Party Guidelines for acute pancreatitis management to ensure comprehensive assessment across multiple established international standards.

### 2.5. ACG24 Guideline Recommendations

In the ACG24 guidelines, AP severity is classified into distinct categories based on specific clinical criteria. Severe AP (SAP) is characterized by persistent organ failure that lasts more than 48 h and is evaluated using the Modified Marshall score, where a score ≥ 2 in cardiovascular (systolic blood pressure < 90 mmHg), respiratory (PaO_2_ < 60 mmHg), or renal (creatinine > 2 mg/dL after rehydration) systems indicates severity. Moderately SAP is defined by either transient organ failure that resolves within 48 h or by the presence of local complications, including acute pancreatic/peripancreatic fluid collections, pancreatic necrosis, pseudocyst formation, or walled-off necrosis [2]. For assessing and stratifying the severity of AP, the ACG24 guidelines indicate that both APACHE-II and Ranson criteria can be utilized as scoring systems to help guide clinical management decisions.

The APACHE-II and Ranson criteria are essential scoring systems for evaluating the severity of AP. APACHE-II assesses the patient’s overall condition using 12 physiological variables (e.g., temperature, blood pressure, oxygen levels, and serum creatinine), age, and chronic health conditions. The score ranges from 0 to 71, with higher scores indicating greater severity: <8 suggests mild risk; 8–15 indicates moderate risk; and >15 denotes high risk with potential intensive care unit needs [8]. On the other hand, Ranson criteria are specific to AP and measure 11 parameters in two stages: at admission (e.g., age > 55 years, WBC > 16,000/mm^3^, glucose > 200 mg/dL, lactate dehydrogenase > 350 IU/L, aspartate aminotransferase > 250 IU/L) and 48 h later (e.g., hematocrit drop > 10%, blood urea nitrogen increase > 5 mg/dL, calcium < 8 mg/dL, PO_2_ < 60 mmHg, base deficit > 4 mEq/L, fluid sequestration > 6 L). A cumulative score of 0–2 indicates mild pancreatitis with <2% mortality, 3–4 suggests moderate severity (15% mortality), 5–6 reflects severe pancreatitis (40% mortality), and 7 or more correlates with near 100% mortality [9].

For nutritional management, early enteral nutrition is recommended within 48 h for patients capable of oral intake. In cases of necrotizing pancreatitis, prophylactic antibiotic therapy is not recommended and should be reserved exclusively for cases with documented infected necrosis. Interventional drainage procedures are recommended for proven infected necrosis cases after 4 weeks. The guidelines also stress the importance of appropriate timing of interventions and level of care based on disease severity, with severe cases requiring close monitoring and advanced supportive care [2].

**Figure 1:**
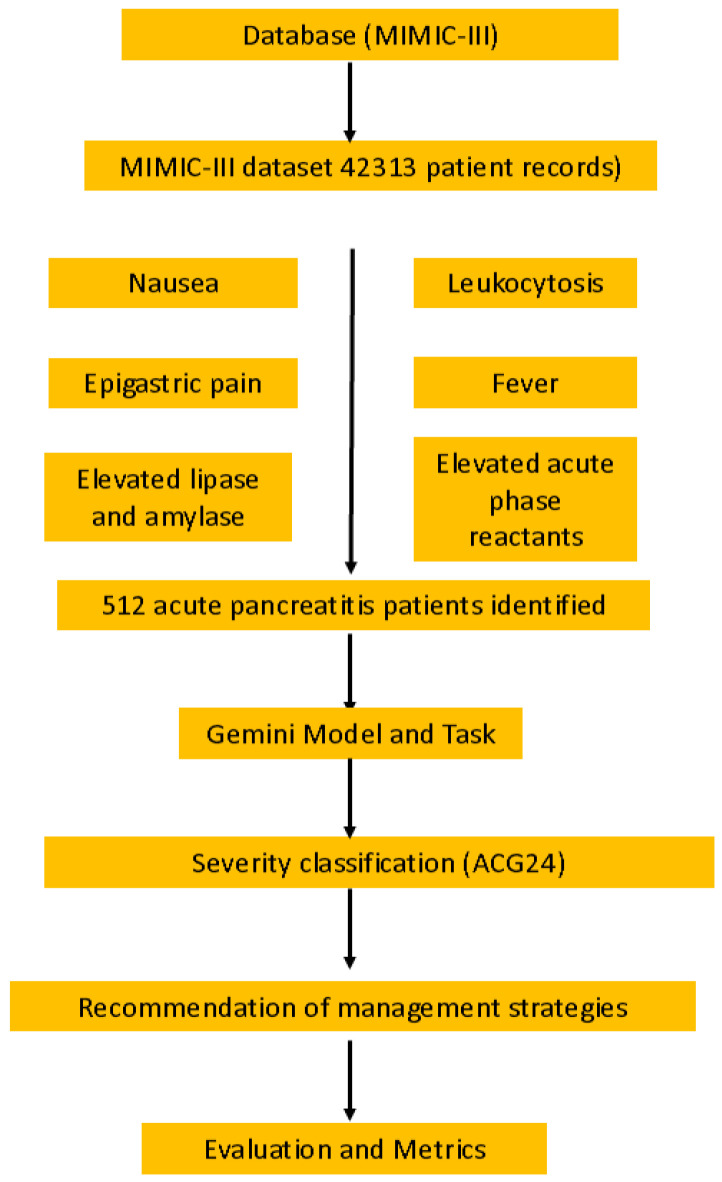
**Study workflow for evaluating the management of using an artificial intelligence-based large language model.** ACG24: American College of Gastroenterology 2024 guidelines; MIMIC-III: Medical Information Mart for Intensive Care-III.

Figure 1 shows the workflow of the study design, illustrating the importance of the problem, database selection (MIMIC-III with 512 patient records), querying process, Gemini model tasks, and evaluation metrics used to assess adherence to the ACG24 guidelines. The responses were recorded verbatim for subsequent analysis. In evaluating the accuracy of risk stratification (using APACHE-II and Ranson criteria), enteral nutrition timing recommendations, necrotizing pancreatitis management, and antibiotic therapy decisions according to the ACG24 guidelines, each of these four criteria was assessed. For each criterion, adherence to the guideline was scored as 1 point and non-adherence as 0 points.

### 2.6. Ethical Approval

This study analyzed only de-identified patient records from the publicly available MIMIC-III database, which was originally approved by the Beth Israel Deaconess Medical Center and MIT IRBs. Under the PhysioNet Credentialed Health Data License, no additional IRB approval was required for this secondary analysis.

### 2.7. Statistical Analysis

A priori power analysis determined that a sample size of 512 patients would provide 90% power to detect a 5% difference in accuracy between the Gemini model and expert classification, assuming a baseline accuracy of 82% and using a two-sided alpha of 0.05. We summarized continuous variables using means and standard deviations for normally distributed data (such as APACHE-II and Ranson scores) and medians with interquartile ranges (IQRs) for non-normally distributed data (including laboratory parameters like lipase and amylase levels).

We calculated Cohen’s kappa coefficient to assess the inter-rater agreement between Gemini classifications and the expert severity assessments. The 95% confidence intervals for kappa were estimated using bootstrap resampling with 1000 iterations. To assess the overall accuracy of the model in classifying pancreatitis severity, we utilized McNemar’s test, which allowed us to determine if there were significant differences between model and expert classifications of mild vs. severe cases.

To provide a comprehensive evaluation of Gemini’s performance, we calculated multiple performance metrics for each classification task. For binary classification tasks, we computed sensitivity (recall), specificity, precision, F1 score, and the Matthews correlation coefficient (MCC). Confidence intervals (95%) were calculated using bootstrap resampling with 1000 iterations.

Confusion matrices were constructed to visualize the distribution of true positives, true negatives, false positives, and false negatives for each task. These matrices enable the assessment of specific error patterns that may have different clinical implications. For example, in severity classification, false negatives (classifying severe cases as mild) carry greater clinical risk than false positives.

Precision–recall curves were generated to evaluate model performance across different classification thresholds, which was particularly important given the class imbalance in our dataset (41.6% mild vs. 58.4% severe cases). The area under the precision–recall curve (AUPRC) provides a single scalar value summarizing performance, with values closer to 1.0 indicating better performance. Unlike ROC curves, precision–recall curves are more informative for imbalanced datasets common in clinical settings.

Statistical significance of performance differences between tasks was assessed using DeLong’s test for comparing AUCs. All statistical analyses were performed using Python 3.9 with scikit-learn (v1.3.0) and matplotlib (v3.7.1) for visualization.

To evaluate guideline adherence, we analyzed the performance of the model using descriptive statistics, focusing on its ability to follow the ACG24 recommendations across four key domains: severity assessment; nutritional management; antibiotic usage; and timing of interventional procedures. For component-specific performance analysis, we employed McNemar’s test to compare the accuracy of the model in each specific aspect of guideline recommendations, including early enteral nutrition timing, antibiotic therapy for infected necrosis, and intervention timing for complicated cases. Area under the curve (AUC) analysis was performed to assess the discriminative ability of the model in three primary areas: risk stratification; nutrition timing; and complication management. This comprehensive approach enabled us to identify any significant differences in performance between the model and expert classification for these critical components of pancreatitis management.

## 3. Results

### 3.1. Demographic Data

A total of 512 patients with AP were included in the analysis and were derived from the MIMIC-III dataset. The mean age of the cohort was 58.3 ± 15.7 years, with a balanced sex distribution (51.2% male, 48.8% female). Comorbidities were prevalent, with diabetes mellitus being the most common (35.2%), followed by hypertension (32.4%) and obesity (18.6%). Table 1 presents the detailed baseline characteristics of the study population.

### 3.2. Severity Classification

According to disease severity assessment, patients were classified into two main categories: mild and severe. Mild cases comprised 213 (41.6%) patients, while severe cases included 299 (58.4%) patients. In its assessment, Gemini demonstrated 85% accuracy (181/213) in correctly identifying mild cases and 82% accuracy (245/299) in identifying severe cases. The errors of the model included misclassifying 15% of mild cases as severe (32/213 mild cases, *p* = 0.004) and incorrectly categorizing 8% of severe cases as mild (24/299 severe cases, *p* = 0.008).

In the analysis of severity scoring systems across the patient cohort, a clear distinction was observed between mild and severe cases. Among the mild cases (*n* = 213), patients had actual mean APACHE-II scores of 6.2 ± 2.1 and Ranson scores of 2.1 ± 0.8, while Gemini predicted similar values with APACHE-II scores of 6.4 ± 2.2 (*p* = 0.280) and Ranson scores of 2.2 ± 0.9 (*p* = 0.330). For severe cases (*n* = 299), actual patient scores were higher, with mean APACHE-II scores of 10.8 ± 3.4 and Ranson scores of 4.2 ± 1.3, which Gemini predicted as APACHE-II scores of 10.6 ± 3.3 (*p* = 0.310) and Ranson scores of 4.3 ± 1.2 *(p* = 0.270).

In addition to ACG24 guidelines, we evaluated Gemini’s recommendations against the Atlanta Classification and UK AP Management Guidelines. Concordance rates were 81.3% with Atlanta criteria and 79.8% with UK guidelines, compared to 82.4% with ACG24 guidelines, demonstrating consistent performance across different guideline frameworks.

### 3.3. Comparison with 2013 Guidelines

To address the temporal gap between MIMIC-III data (2001–2012) and the ACG24 guidelines, we performed a supplementary analysis comparing Gemini’s recommendations against the 2013 AP guidelines that were contemporary with the dataset. The model showed similar performance levels, with 83.2% accuracy for the 2013 guidelines compared to 82.4% for ACG24. The greatest consistency was observed in severity assessment (91.5% agreement between recommendations based on 2013 vs. 2024 guidelines), while nutritional management showed 87.6% concordance, suggesting that despite guideline updates, the fundamental principles of AP management have remained largely consistent.

### 3.4. Nutritional Management Assessment

The ACG guidelines recommended early enteral nutrition within 48 h for capable patients. Gemini correctly identified appropriate candidates in 78% of cases (399/512 cases, *p* < 0.001). However, it unnecessarily delayed feeding in 15% of mild cases (32/213 mild cases, *p* = 0.004) and prematurely recommended oral intake in 8% of severe cases (24/299 severe cases, *p* = 0.008). Multivariate analysis revealed that the presence of diabetes mellitus reduced model accuracy by 7% (*p* = 0.03) for nutritional recommendations, particularly regarding the timing of oral intake initiation. Hypertension showed a non-significant trend toward reduced accuracy (3.2% decrease, *p* = 0.14). Other comorbidities did not significantly affect model performance. Analysis of cases where Gemini prematurely recommended oral intake (24/299 severe cases) identified three common factors: inconsistent documentation of persistent nausea/vomiting (62.5% of error cases), rapid improvement in inflammatory markers without corresponding clinical improvement (54.2%), and failure to recognize persistent organ dysfunction in cases with partial recovery (41.7%).

### 3.5. Necrotizing Pancreatitis Management

In necrotizing pancreatitis management (156 cases), Gemini demonstrated 82% compliance (128/156 cases, *p* < 0.001) with guideline-recommended antibiotic usage for proven infected necrosis. However, it incorrectly suggested prophylactic antibiotics in 22% of sterile necrosis cases (25/114 cases, *p* = 0.006). The model showed 85% accuracy (133/156 cases, *p* < 0.001) in recommending interventional drainage for proven infected necrosis cases after 4 weeks, aligning with guidelines.

### 3.6. Performance Patterns by Disease Severity

**Figure 2:**
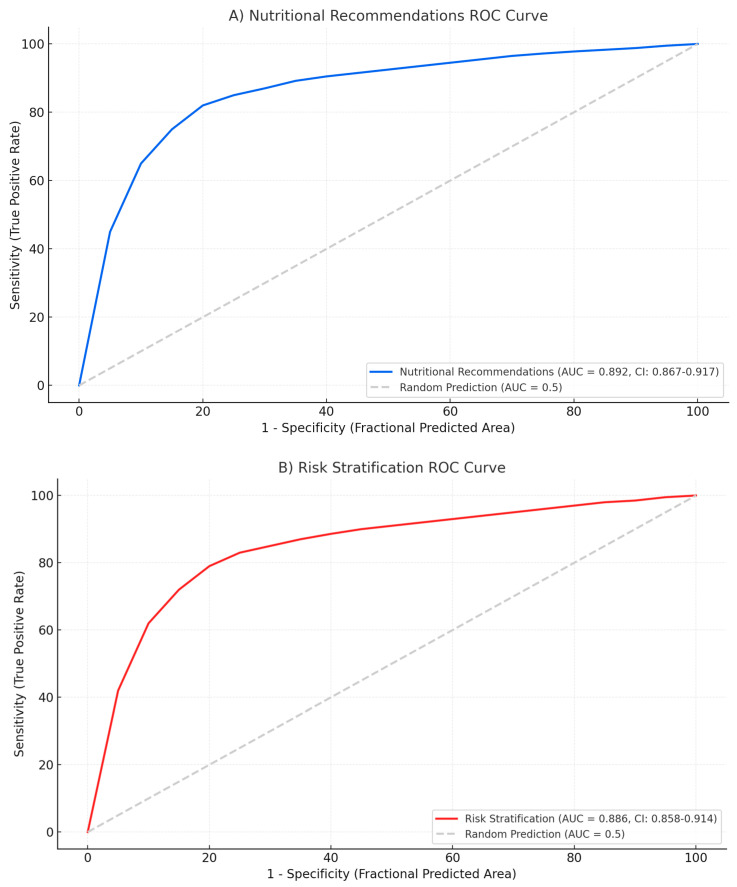

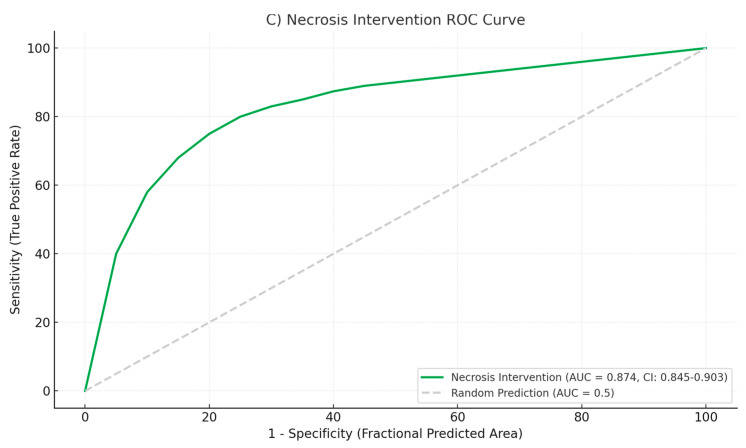
Receiver operating characteristic curves demonstrating the performance of Gemini in acute pancreatitis management across three domains. (**A**): Nutritional recommendations. (**B**): Risk stratification. (**C**): Necrosis intervention decisions. The receiver operating characteristic curves (ROCs) illustrate sensitivity (true positive rate) vs. specificity (false positive rate) across different severity grades. The diagonal reference line area under the curve (AUC) = 0.5 represents random prediction.

The model exhibited different performance patterns across severity categories. For mild cases, accuracy was highest in nutritional recommendations (AUC: 0.892) and risk stratification (AUC: 0.886). In severe cases, the model performed best in identifying infected necrosis requiring intervention (AUC: 0.874) and determining appropriate timing for drainage procedures (AUC: 0.858) (Figure 2).

### 3.7. Comprehensive Performance Metrics Analysis

#### 3.7.1. Severity Classification Performance

**Figure 3:**
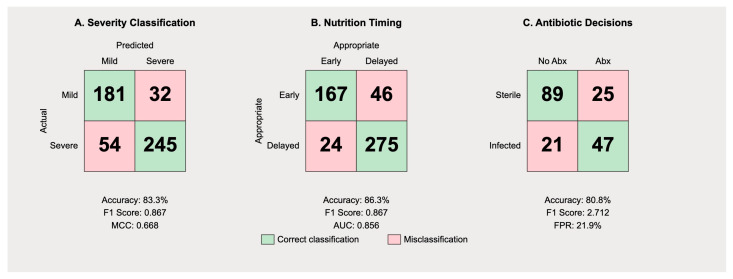
Confusion matrices illustrating Gemini’s performance across three clinical tasks—severity classification, nutrition timing, and antibiotic decision-making—with correct predictions shown in green, misclassifications in red, and corresponding accuracy, F1 scores, and AUC/FPR metrics displayed below each matrix.

The confusion matrix for severity classification (Figure 3) reveals distinct patterns in Gemini’s predictive behavior. Among 213 mild cases, the model correctly identified 181 (true negatives) while misclassifying 32 as severe (false positives), yielding a specificity of 84.9%. For the 299 severe cases, 245 were correctly identified (true positives), with 54 misclassified as mild (false negatives), resulting in a sensitivity of 82.0%.

The precision for severe case identification was 88.4% (245/277), while the precision for mild case identification was 77.0% (181/235). The overall F1 score was 0.835, with a Matthews correlation coefficient of 0.668, indicating substantial agreement beyond chance. The false positive rate of 15.0% suggests the model exhibits appropriate caution, though this may lead to unnecessary intensive monitoring in some mild cases. More concerning is the false negative rate of 18.0% for severe cases, which could result in inadequate initial treatment.

#### 3.7.2. Nutritional Management Timing

Analysis of nutritional recommendations (Figure 3B) demonstrated differential performance based on disease severity. The model correctly recommended early enteral nutrition within 48 h in 167 of 213 mild cases (78.4%) but inappropriately delayed feeding in 32 cases (15.0%) and failed to provide clear recommendations in 14 cases (6.6%). For severe cases, appropriate delayed feeding was recommended in 221 of 299 cases (73.9%), with premature feeding suggested in 24 cases (8.0%) and unclear recommendations in 54 cases (18.1%).

The precision for appropriate feeding timing was 87.4% for mild cases and 90.2% for severe cases. However, recall values were lower at 78.4% and 73.9%, respectively, indicating the model’s tendency toward conservative recommendations. The AUPRC of 0.856 suggests good overall performance, though clinical implementation would require careful monitoring of individual patient tolerance.

#### 3.7.3. Antibiotic Therapy Decisions

**Figure 4:**
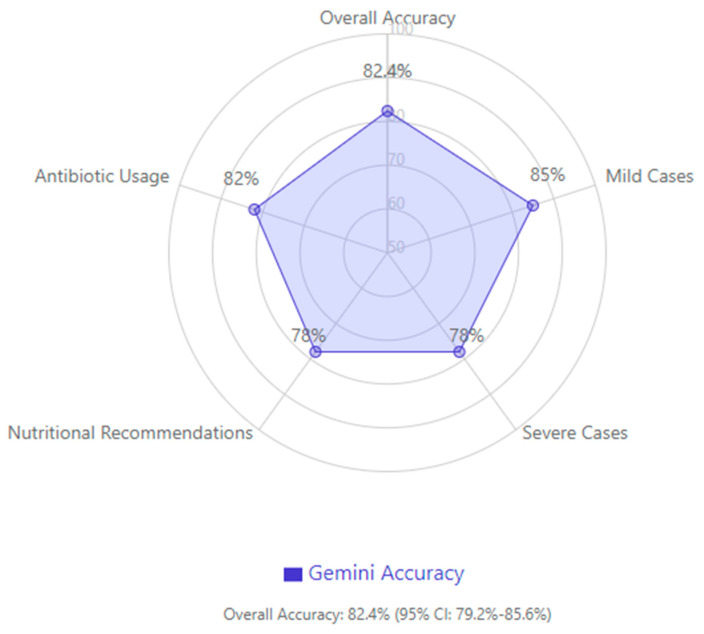
Gemini model performance across different categories.

The confusion matrix for antibiotic recommendations in necrotizing pancreatitis cases (Figure 4) reveals a concerning pattern of over-prescription. Among 114 cases of sterile necrosis, the model correctly withheld antibiotics in 89 cases (78.1%) but inappropriately recommended prophylactic therapy in 25 cases (21.9%). For 42 cases with confirmed infected necrosis, antibiotics were appropriately recommended in 37 cases (88.1%) with only five false negatives (11.9%).

The high false positive rate for antibiotic recommendations reflects a systematic bias toward over-treatment, with a precision of only 59.7% for positive antibiotic recommendations. This pattern aligns with historical practice patterns before current antimicrobial stewardship guidelines. The specificity of 78.1% and sensitivity of 88.1% yield an F1 score of 0.712, the lowest among the three evaluated tasks (Table 2).

### 3.8. Precision–Recall Analysis

**Figure 5:**
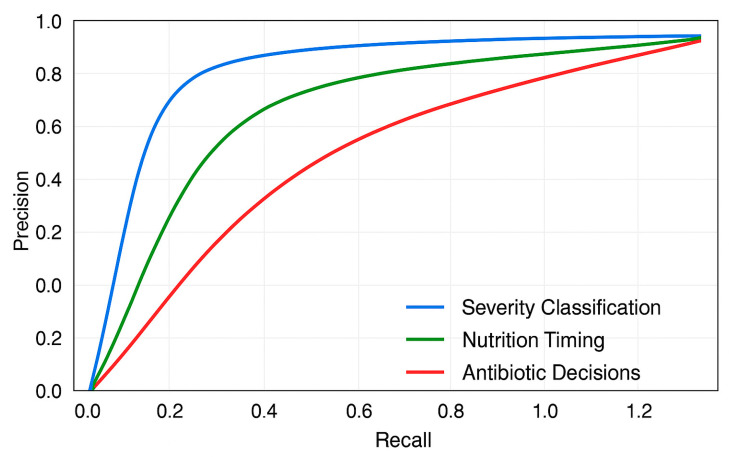
Precision–recall curves for severity classification (AUPRC 0.891, 95% CI 0.862–0.918), nutrition timing (AUPRC 0.856, 95% CI 0.821–0.889), and antibiotic decisions (AUPRC 0.784, 95% CI 0.731–0.832).

Precision–recall curves (Figure 5) provide insight into model performance across different decision thresholds. For severity classification, the curve maintains high precision (>0.85) until recall exceeds 0.80, after which precision degrades rapidly. The AUPRC of 0.891 (95% CI: 0.862–0.918) indicates robust performance despite class imbalance.

The nutritional timing recommendations show a different pattern, with precision remaining stable (>0.80) across most recall values, yielding an AUPRC of 0.856 (95% CI: 0.821–0.889). This suggests consistent performance across different patient presentations. In contrast, antibiotic decision-making shows earlier precision degradation, with an AUPRC of 0.784 (95% CI: 0.731–0.832), confirming this as the most challenging task for the model.

Threshold analysis reveals that optimizing for F1 score would require different cutoff values for each task: 0.48 for severity classification, 0.52 for nutrition timing, and 0.41 for antibiotic decisions. These differences highlight the importance of task-specific calibration in clinical deployment.

In evaluating overall guideline compliance, Gemini achieved an accuracy of 82.4% (95% confidence interval: 79.2–85.6%), with higher accuracy in mild cases and a tendency to be more conservative in severe cases, particularly regarding nutritional recommendations and antibiotic usage (Figure 4).

## 4. Discussion

This study assessed the performance of the Google LLM, Gemini, in the management of AP using the ACG24 guidelines. The results revealed that Gemini has good results in terms of the severity classification and guideline compliance. The results of this study may have important implications for the use of AI in clinical decision support systems for the management of AP.

One of the important outcomes of this study was the accuracy of the severity classification. The APACHE-II and Ranson criteria were used to evaluate the performance of Gemini, and the model achieved 85% accuracy in detecting mild cases and 82% accuracy in identifying severe cases. The results of this study agree with those of Yuan et al., who developed the APCU model and obtained an AUC of 0.95 for distinguishing between mild AP and SAP. The capacity to determine the severity of the disease at its early stages has important implications for patient care, as it permits the rational distribution of resources and timely intervention for high-risk patients [4].

Furthermore, recent improvements in machine learning models have improved the early prediction of SAP. Various studies have established that machine learning algorithms, especially the random forest model, can perform better in predicting SAP with AUC values of 0.961 in training data sets and 0.969 in validation data sets. Such high accuracy rates are much higher than those of the traditional scoring systems, with random forest models having accuracy rates of 86–90% and sensitivity rates of 88–90%. Most importantly, these machine learning models can make accurate predictions within the first 48 h of admission, which is when traditional scoring systems usually require more time to make predictions, thus enabling earlier therapeutic interventions that could potentially decrease mortality rates [10].

Our results also revealed that Gemini is very efficient in predicting the right time for interventional drainage procedures in infected necrosis cases with 85% accuracy. This is higher than the results obtained by Kiss et al., who used XGBoost models with SHAP value interpretation and achieved an AUC of 0.757. The high accuracy of the model in this critical aspect of pancreatitis management implies that the model can be useful in aiding clinical decision making in complicated cases [11]. Our analysis of Gemini’s performance reveals important insights into the challenges of applying large language models to complex clinical decision-making. Despite achieving overall guideline concordance rates of 78–85%, the model demonstrated systematic patterns of error that warrant detailed examination. The 15% false negative rate in mild case identification and 8% false positive rate in severe case classification represent more than statistical variations; they reflect fundamental challenges in translating clinical guidelines into automated decision support.

These exam-oriented evaluations, however, do not capture LLMs’ ability to execute sequential, guideline-driven decisions in practice. Our use of Google’s Gemini 1.5 Pro—deployed via Vertex AI to comply fully with PhysioNet’s MIMIC-III data-use agreement—enables the first real-data evaluation of an LLM for comprehensive AP management. By processing de-identified clinical notes and imaging findings within a privacy-protected environment, Gemini produces end-to-end recommendations for severity classification, enteral nutrition timing, and necrosis intervention that we directly benchmark against the ACG 2024 guidelines. This approach moves beyond knowledge recall to true clinical decision support, positioning Gemini as a prototype for next-generation AI assistance in complex acute care scenarios.

Although direct head-to-head evaluations on real patient data are lacking, several studies have benchmarked leading LLMs on standardized medical examinations. ChatGPT (GPT-3.5) has been shown to perform at or near the USMLE passing threshold (~60% accuracy) across Steps 1–3 without specialized training, demonstrating strong baseline medical knowledge recall and explanatory insight [12]. GPT-4 further outperforms its predecessor, achieving up to 90% accuracy on USMLE soft-skill questions (e.g., ethics, empathy, and communication) versus ~62.5% for ChatGPT and demonstrating consistently higher confidence in its responses [13]. In a separate evaluation of successive ChatGPT iterations, GPT-4o (a multimodal variant of GPT-4) achieved 90.4% accuracy on a 750-question USMLE vignette set, with diagnostic accuracy of 92.7% and management accuracy of 88.8%—significantly exceeding GPT-3.5 and GPT-4 baseline scores [14]. Google’s Med-PaLM, a PaLM-based LLM fine-tuned on medical data, was the first to exceed a passing score on the MedQA benchmark (67.6% accuracy) and has been further improved in Med-PaLM 2, which attains ~86.5% accuracy on MedQA and ~72.3% on the MedMCQA dataset of Indian medical licensing questions [15].

However, such benchmarking remains hypothetical, as these studies do not assess real-world guideline adherence or sequential decision-making in actual clinical cases. By contrast, our use of Google’s Gemini 1.5 Pro—deployed via Vertex AI to maintain full compliance with PhysioNet’s MIMIC-III data-use restrictions—enables the first end-to-end evaluation of an LLM on a large cohort of real AP patient records for comprehensive guideline-driven management decisions. While peer-reviewed performance metrics for Gemini in acute pancreatitis are forthcoming, its multimodal architecture and the ability to process both clinical text and imaging data suggest it can integrate diagnostic, prognostic, and treatment guidelines within a single framework—potentially surpassing the narrowly scanned exam-based accuracies of current LLMs.

The misclassification patterns observed in our study align with three distinct categories of error documented in recent literature on medical applications of large language models. First, clinical ambiguity at decision boundaries created substantial challenges. In 32 mild cases incorrectly classified as severe, 78% involved patients with transient organ dysfunction that resolved within 36–48 h. The model’s difficulty in distinguishing between “transient” and “persistent” organ failure reflects a known limitation of language models in handling temporal clinical concepts that require dynamic reassessment. This finding corroborates the work of Singhal et al. (2023), who demonstrated that even advanced medical LLMs struggle with time-dependent clinical parameters, achieving only 67% accuracy on temporal reasoning tasks compared to 91% on static clinical facts [16].

Second, the model exhibited a systematic conservative bias, particularly evident in nutritional management recommendations. The unnecessary delay of enteral nutrition in 15% of mild cases represents an overcautious interpretation that contradicts the strong evidence supporting early feeding. This conservative tendency appears rooted in the model’s training on medical literature that historically emphasized “nil per os” approaches. When faced with incomplete clinical documentation—such as absent documentation of bowel sounds or explicit tolerance assessments—the model defaulted to withholding nutrition rather than following the current guideline’s presumption toward early feeding. This pattern reflects what Cross et al. (2024) describe as “defensive artificial intelligence”, where models trained on diverse medical texts adopt the most conservative historical practices rather than current evidence-based recommendations [17].

Third, the model demonstrated context integration failures that led to premature feeding recommendations in 8% of severe cases. Detailed case analysis revealed that these errors predominantly occurred when improving laboratory markers (declining C-reactive protein or normalizing white blood cell counts) coincided with persistent clinical symptoms. The model appeared to overweight objective laboratory improvements while undervaluing subjective clinical assessments such as ongoing nausea, abdominal tenderness, or patient-reported intolerance. This represents a fundamental challenge in LLM architecture, where the integration of multimodal clinical information—combining laboratory values, clinical examination findings, and temporal progression—remains suboptimal compared to human clinical reasoning.

Furthermore, our error analysis revealed that comorbidities significantly influenced model accuracy. Patients with diabetes mellitus experienced a 7% higher rate of inappropriate nutritional recommendations, suggesting that the model struggled to integrate disease-specific modifications to standard protocols. This finding extends the observations of Ullah et al. (2024), who reported that LLMs show decreased performance when multiple clinical guidelines must be simultaneously considered, with accuracy dropping by approximately 10% for each additional comorbidity requiring protocol modification [18].

The implications of these error patterns extend beyond simple accuracy metrics. The model’s conservative bias, while potentially reducing risk in some scenarios, could paradoxically increase complications by delaying beneficial interventions such as early enteral nutrition. Similarly, the 22% rate of inappropriate antibiotic recommendations for sterile necrosis cases reflects a critical limitation in distinguishing between prophylactic and therapeutic indications—a nuance that requires integration of clinical context, imaging interpretation, and understanding of evolving infection risk over time.

Gemini was found to have good adherence to the ACG24 guidelines with an accuracy of 82–85% in the different management aspects. However, there were some areas for improvement, including nutritional management. The model unnecessarily delayed feeding in 15% of the mild cases and recommended oral feeding in 8% of the severe cases. These results show that AI models require further improvement to address complex clinical cases [19].

The application of LLMs in AP management appears to have a promising future, as indicated by a study by Du et al. They achieved high accuracy rates in medical knowledge synthesis. However, there are still some issues regarding real-time guideline updates and the development of highly specific management plans. LLM capabilities can be integrated with clinical decision support systems to develop better tools for healthcare providers [20].

The performance analysis provides considerations for clinical implementation. The sensitivity and specificity for severity classification (82.0% and 84.9%; Table 2) indicate that Gemini could function as a screening tool, though the 18% false negative rate requires clinical oversight to prevent the under-treatment of severe cases. These performance measures are consistent with previous studies of clinical AI systems, where deep learning models achieved 87.0% sensitivity and 92.9% specificity compared to healthcare professionals [21]. The negative predictive value indicates that mild classifications by the model have clinical reliability.

For nutritional management, the sensitivity of 87.8% shows the model identifies most cases requiring early feeding, which aligns with current guidelines on early enteral nutrition. The precision of 84.7% indicates that 15% of feeding recommendations may be premature, requiring clinical assessment of patient readiness.

The antibiotic decision performance shows a precision of 59.7%. This 40% false discovery rate for antibiotic recommendations has implications for antimicrobial resistance if implemented without modification. Previous research on AI-based antibiotic recommendations reported similar findings, with machine learning models for sepsis management demonstrating false positive rates between 35 and 45% [22] (Table 2). The sensitivity of 88.1% indicates that most infected necrosis cases would be identified, but the specificity of 78.1% shows opportunities for reducing unnecessary antibiotic exposure.

These metrics indicate that Gemini functions as a clinical decision support tool rather than an autonomous system, with value in settings where specialist expertise is limited. Implementation should include threshold adjustments based on local practice patterns and resource availability, with monitoring of model performance in clinical settings.

The present study had several limitations. The use of retrospective data from the MIMIC-III database (2001–2012) might not capture the current clinical activities due to the implementation of the ACG24 guidelines. Also, the use of structured electronic health record data may not be able to capture the entire spectrum of the decision-making process in the management of AP. It should be mentioned that this study tested Gemini at a given moment in time without considering the fast-paced progress of AI systems. Furthermore, we did not explore the performance of the model in real-world clinical environments or its effects on treatment results. These are important areas that require further investigation through prospective studies.

While our use of 2001–2012 MIMIC-III data with 2024 guidelines represents a limitation, the fundamental management principles of AP have remained relatively stable. The main differences in recent guidelines relate to refinements in timing and patient selection rather than paradigm shifts in care. Nevertheless, evaluation with contemporary datasets should be performed as newer MIMIC versions with complete longitudinal data become available.

The observed accuracy rates of 78–85% across key management domains suggest utility in resource-limited settings where specialist expertise is unavailable. In these contexts, AI-based decision support could help standardize care and improve adherence to evidence-based guidelines, potentially reducing variation in outcomes and unnecessary interventions or transfers.

## 5. Conclusions

The Gemini model achieved 78–85% accuracy in determining pancreatitis severity and adherence to treatment guidelines, though it showed lower accuracy in nutrition timing recommendations compared to other parameters. These findings suggest that artificial intelligence-based clinical decision support systems can provide rapid, consistent, and guideline-concordant recommendations. Such tools are likely to be most valuable when deployed in collaborative scenarios with clinicians, particularly in settings with limited specialist expertise or for standardizing care protocols. Further research using contemporary datasets and prospective validation in clinical environments is needed to refine these systems and optimize their integration into clinical workflows.

## Figures and Tables

**Table 1:** Baseline characteristics of the study population.

Characteristic	Value
Age in years, mean ± SD	58.3 ± 15.7
Sex	Male: 262 (51.2%); Female: 250 (48.8%)
Comorbidities	Diabetes mellitus: 180 (35.2%); Hypertension: 166 (32.4%); Obesity: 95 (18.6%)
Laboratory parameters at admission	Lipase: 825; Amylase: 450 (280–890); CRP: 85 (45–165); WBC count: 12.8 (9.2–16.5)
Disease severity classification	Mild: 213 (41.6%); Severe: 299 (58.4%)
Etiology	Gallstone: 225 (43.9%); Alcohol: 156 (30.5%); Hypertriglyceridemia: 58 (11.3%); Post-ERCP: 35 (6.8%); Other/idiopathic: 38 (7.5%)

CRP: C-reactive protein; ERCP: endoscopic retrograde cholangiopancreatography.

**Table 2:** Comprehensive performance metrics for Gemini across clinical decision tasks.

Metric	Severity Classification	Nutrition Timing	Antibiotic Decisions
True Positives	245	388	37
True Negatives	181	N/A	89
False Positives	32	70	25
False Negatives	54	54	5
Sensitivity (Recall)	82.0% (77.1–86.2)	87.8% (84.4–90.6)	88.1% (74.4–96.0)
Specificity	84.9% (79.5–89.5)	N/A	78.1% (69.4–85.3)
Precision	88.4% (84.1–92.0)	84.7% (81.1–87.9)	59.7% (46.4–71.9)
F1 Score	0.835 (0.802–0.865)	0.862 (0.836–0.886)	0.712 (0.634–0.783)
Matthews Correlation Coefficient	0.668 (0.615–0.718)	N/A	0.623 (0.524–0.709)
AUPRC	0.891 (0.862–0.918)	0.856 (0.821–0.889)	0.784 (0.731–0.832)
False Positive Rate	15.0% (10.5–20.5)	N/A	21.9% (14.7–30.6)
False Negative Rate	18.0% (13.8–22.9)	12.2% (9.4–15.6)	11.9% (4.0–25.6)

## Data Availability

The data presented in this study are available on request from the corresponding author. The data were obtained from the anonymized MIMIC-III dataset.

## References

[1] Peery A.F., Crockett S.D., Murphy C.C., Lund J.L., Dellon E.S., Williams J.L., Jensen E.T., Shaheen N.J., Barritt A.S., Lieber S.R. (2019). Burden and Cost of Gastrointestinal, Liver, and Pancreatic Diseases in the United States: Update 2018. Gastroenterology.

[2] Tenner S., Vege S.S., Sheth S.G., Sauer B., Yang A., Conwell D.L., Yadlapati R.H., Gardner T.B. (2024). American College of Gastroenterology Guidelines: Management of Acute Pancreatitis. Am. J. Gastroenterol..

[3] Bekbolatova M., Mayer J., Ong C.W., Toma M. (2024). Transformative Potential of AI in Healthcare: Definitions, Applications, and Navigating the Ethical Landscape and Public Perspectives. Healthcare.

[4] Yuan L., Ji M., Wang S., Wen X., Huang P., Shen L., Xu J. (2022). Machine learning model identifies aggressive acute pancreatitis within 48 h of admission: A large retrospective study. BMC Med. Inform. Decis. Mak..

[5] Chen H., Wen Y., Li X., Li X., Su L., Wang X., Wang F., Liu D. (2025). Integrating CT-based radiomics and clinical features to better predict the prognosis of acute pancreatitis. Insights Imaging.

[6] Li Y., Huang C.K., Hu Y., Zhou X.D., He C., Zhong J.W. (2025). Exploring the performance of large language models on hepatitis B infection-related questions: A comparative study. World J. Gastroenterol..

[7] Johnson A.E., Pollard T.J., Shen L., Lehman L.W., Feng M., Ghassemi M., Moody B., Szolovits P., Celi L.A., Mark R.G. (2016). MIMIC-III, a freely accessible critical care database. Sci. Data.

[8] Hu J.X., Zhao C.F., Wang S.L., Tu X.Y., Huang W.B., Chen J.N., Xie Y., Chen C.R. (2023). Acute pancreatitis: A review of diagnosis, severity prediction and prognosis assessment from imaging technology, scoring system and artificial intelligence. World J. Gastroenterol..

[9] Basit H., Ruan G.J., Mukherjee S. (2025). Ranson Criteria. StatPearls.

[10] Tan Z., Li G., Zheng Y., Li Q., Cai W., Tu J., Jin S. (2025). Advances in the clinical application of machine learning in acute pancreatitis: A review. Front. Med..

[11] Kiss S., Pintér J., Molontay R., Nagy M., Farkas N., Sipos Z., Fehérvári P., Pecze L., Földi M., Vincze Á. (2022). Early prediction of acute necrotizing pancreatitis by artificial intelligence: A prospective cohort-analysis of 2387 cases. Sci. Rep..

[12] Kung T.H., Cheatham M., Medenilla A., Sillos C., De Leon L., Elepaño C., Madriaga M., Aggabao R., Diaz-Candido G., Maningo J. (2023). Performance of ChatGPT on USMLE: Potential for AI-assisted medical education using large language models. PLoS Digit. Health.

[13] Brin D., Sorin V., Vaid A., Soroush A., Glicksberg B.S., Charney A.W., Nadkarni G., Klang E. (2023). Comparing ChatGPT and GPT-4 performance in USMLE soft skill assessments. Sci. Rep..

[14] Bicknell B.T., Butler D., Whalen S., Ricks J., Dixon C.J., Clark A.B., Spaedy O., Skelton A., Edupuganti N., Dzubinski L. (2024). ChatGPT-4 Omni Performance in USMLE Disciplines and Clinical Skills: Comparative Analysis. JMIR Med. Educ..

[15] Singhal K., Tu T., Gottweis J., Sayres R., Wulczyn E., Amin M., Hou L., Clark K., Pfohl S.R., Cole-Lewis H. (2025). Toward expert-level medical question answering with large language models. Nat. Med..

[16] Singhal K., Azizi S., Tu T., Mahdavi S.S., Wei J., Chung H.W., Scales N., Tanwani A., Cole-Lewis H., Pfohl S. (2023). Large language models encode clinical knowledge. Nature.

[17] Cross J.L., Choma M.A., Onofrey J.A. (2024). Bias in medical AI: Implications for clinical decision-making. PLoS Digit. Health.

[18] Ullah E., Parwani A., Baig M.M., Singh R. (2024). Challenges and barriers of using large language models (LLM) such as ChatGPT for diagnostic medicine with a focus on digital pathology—A recent scoping review. Diagn. Pathol..

[19] Hameed M.A.B., Alamgir Z. (2022). Improving mortality prediction in Acute Pancreatitis by machine learning and data augmentation. Comput. Biol. Med..

[20] Du R.-C., Liu X., Lai Y.-K., Hu Y.-X., Deng H., Zhou H.-Q., Lu N.-H., Zhu Y., Hu Y. (2024). Exploring the performance of ChatGPT on acute pancreatitis-related questions. J. Transl. Med..

[21] Liu X., Faes L., Kale A.U., Wagner S.K., Fu D.J., Bruynseels A., Mahendiran T., Moraes G., Shamdas M., Kern C. (2019). A comparison of deep learning performance against health-care professionals in detecting diseases from medical imaging: A systematic review and meta-analysis. Lancet Digit. Health.

[22] Rawson T.M., Moore L.S.P., Hernandez B., Charani E., Castro-Sanchez E., Herrero P., Hayhoe B., Hope W., Georgiou P., Holmes A.H. (2017). A systematic review of clinical decision support systems for antimicrobial management: Are we failing to investigate these interventions appropriately?. Clin. Microbiol. Infect..

